# A Variant of Fibroblast Growth Factor Receptor 2 (Fgfr2) Regulates Left-Right Asymmetry in Zebrafish

**DOI:** 10.1371/journal.pone.0021793

**Published:** 2011-07-01

**Authors:** Da-Wei Liu, Chia-Hao Hsu, Su-Mei Tsai, Chung-Der Hsiao, Wen-Pin Wang

**Affiliations:** 1 Institute of Medical Sciences, Tzu-Chi University, Hualien, Taiwan; 2 Department of Molecular Biology and Human Genetics, Tzu-Chi University, Hualien, Taiwan; 3 Department of Bioscience Technology, Chung Yuan Christian University, Chung-Li, Taiwan; 4 Center for Nanotechnology, Chung Yuan Christian University, Chung-Li, Taiwan; Laboratoire Arago, France

## Abstract

Many organs in vertebrates are left-right asymmetrical located. For example, liver is at the right side and stomach is at the left side in human. Fibroblast growth factor (Fgf) signaling is important for left-right asymmetry. To investigate the roles of Fgfr2 signaling in zebrafish left-right asymmetry, we used splicing blocking morpholinos to specifically block the splicing of *fgfr2b* and *fgfr2c* variants, respectively. We found that the relative position of the liver and the pancreas were disrupted in *fgfr2c* morphants. Furthermore, the left-right asymmetry of the heart became random. Expression pattern of the laterality controlling genes, *spaw* and *pitx2c*, also became random in the morphants. Furthermore, *lefty1* was not expressed in the posterior notochord, indicating that the molecular midline barrier had been disrupted. It was also not expressed in the brain diencephalon. Kupffer's vesicle (KV) size became smaller in *fgfr2c* morphants. Furthermore, KV cilia were shorter in *fgfr2c* morphants. We conclude that the *fgfr2c* isoform plays an important role in the left-right asymmetry during zebrafish development.

## Introduction

The bodies of most adult animals have left-right symmetry. However, some organs are not symmetrical, including the heart, liver, spleen, stomach, and pancreas [1]. When laterality is disrupted, many defects can result, such as abnormal position of organs, skeletal malformation and failure of neural tube closure [2]. Mechanisms involved in the regulation of laterality for various animal species have been identified. These include motor proteins, ion channel, cytoskeleton, serotonin, cell-cell junction, Ca^2+^, and cilia [3]. For example, in the mouse, the leftward movement of fluid at the ventral node, called nodal flow, is the critical process for left-right asymmetry [4]. The nodal flow is generated by the clockwise rotation of nodal cilia. This directional flow causes some morphogens to concentrate at left side of the node and leads to left-right polarization [5], [6].

Many signaling molecules are involved in left-right asymmetry, including Nodal and Sonic hedgehog (SHH) [1], [7], [8]. Signaling of fibroblast growth factors (FGFs) has been shown to regulate Nodal signaling in left-right determination [9], [10]. The expression of *nodal* is suppressed by FGF8 in chicken [9]. In contrast, FGF8 can induce *nodal* expression in mouse [10]. Moreover, FGF signaling triggers secretion of nodal vesicular parcels which carry SHH and retinoic acid [6]. There are 22 FGF ligands in human and mice, and 27 in zebrafish [11], [12]. Four FGF receptors (FGFRs) have been identified, including FGFR1, FGFR2, FGFR3, and FGFR4 in vertebrates. Furthermore, FGFR1, FGFR2, and FGFR3 can be classified into b and c isoforms by alternative splicing. The binding specificity of FGFs with FGFRs is provided by the diversity of the FGF sequence and alternative splicing of FGFRs [13], [14]. Among them, FGFR2b can bind with FGF1, FGF3, FGF7, FGF10 and FGF22; FGFR2c can bind with FGF1, FGF2, FGF4, FGF6, FGF9, FGF17 and FGF18.

In zebrafish, Kupffer's vesicle (KV) is equivalent to the mouse node and is important for left-right development [15]. KV is a fluid-filled ciliated organ. Recent results indicate that most cilia are located on the dorsal side and are distributed along the anterior-posterior axis unequally [16]. Interestingly, unlike the leftward fluid flow in mice, fluid flow generated in the KV of zebrafish has a counter-clockwise rotation [17]. Recently, Fgf signaling regulation of laterality has been reported in zebrafish [18], [19], [20]. In *ace/fgf8* mutant, the asymmetric visceral organs and the proper symmetric craniofacial skeleton are disrupted. Furthermore, the KV morphogenesis is defective in *ace/fgf8* mutant fish [18]. Moreover, Fgf signaling can regulate the length of cilia through the Fgf8/Fgf24-Fgfr1 pathway [20]. The downstream effectors of Fgf signaling, Ier2 and Fibp1, are also identified in the process of KV ciliogenesis [19].

We studied the role of Fgfr2 in liver development (manuscript in preparation). Unexpectedly, we detected that left-right asymmetry of visceral organs was randomized in the different *fgfr2* morphants, especially for the *fgfr2c*. Furthermore, normal heart jogging and looping were disrupted in the morphants. The expression of specific left-sided genes, such as *spaw*, *pitx2c* and *lefty1*, was affected in *fgfr2c* morphants. The expression of *spaw* and *pitx2c* was randomized in left lateral plate mesoderm (LPM) of *fgfr2c* morphants. However, the expression of *lefty1* was absent in most *fgfr2c* morphants. Furthermore, we found that ciliogenesis was defective in *fgfr2c* morphants: the cilia length was shorter in *fgfr2c* morphants. This phenomenon was similar in *fgfr1*, *fgf8,* and *fgf8/fgf24* morphants. These results suggest that Fgfr2c is important in the regulation of left-right asymmetry.

## Results

### Visceral Organ Laterality was Affected by *fgfr2* Inhibition

Fgf signaling pathways have been proposed to regulate liver specification [21]. However, the critical Fgf receptor(s) that participate in this process have not been fully characterized. We found the expression of earliest marker for developing liver, hematopoietically expressed homeobox gene (hhex), was absented in more than half of *fgfr2* morphants (data not shown).

**Figure 1:**
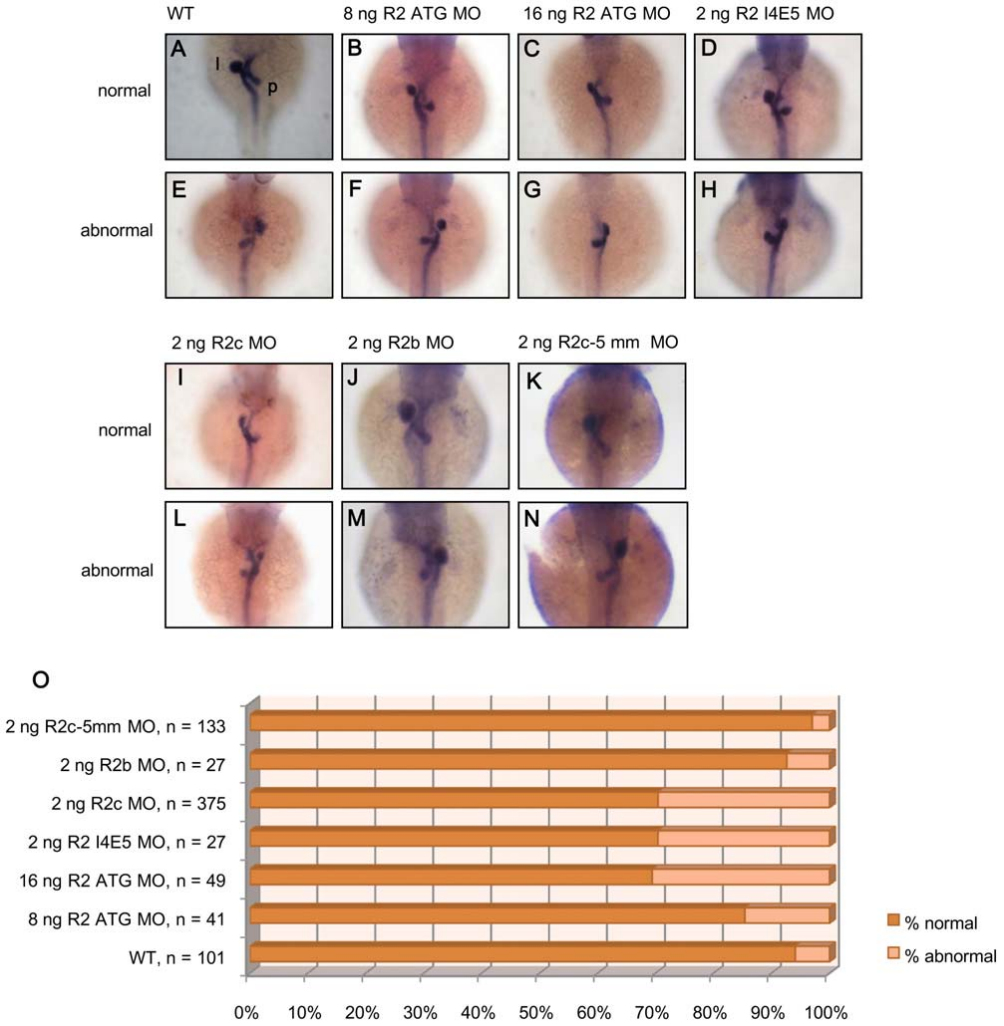
The left-right asymmetry of visceral organs was randomized in *fgfr2* morphants. The expression pattern of *foxA3* in liver (l) and pancreas (p) was shown in wild type, *fgfr2*-ATG morphants, *fgfr2*-I4E5 morphants, *fgfr2c* morphants, *fgfr2b* and *fgfr2c*-5 mm morphants (A∼D, I∼K). Abnormal pattern of reverse visceral organs was also observed in these embryos (E∼H, L∼N). All pictures were dorsal view. The bar charts showed the percentage of left-right asymmetry of visceral organs (O).

Unexpectedly, the disruption of left-right asymmetry in the *fgfr2*-ATG morphants was noticed from the expression pattern of *foxA3* at 48 hours post fertilization (hpf). In 94.1% (*n* = 101) of the wild type embryos, the liver bud was located on the left side and the pancreatic bud was located on the right side in zebrafish (Fig. 1A and 1O). In 5.9% of the wild type embryos, abnormal left-right asymmetry was observed, and the relative locations of liver and pancreas were reversed (Fig. 1E and 1O). In *fgfr2*-ATG morphants, the left-right pattern was affected, and the abnormal percentages increased with dosages (Fig. 1B, C, F, G, and 1O; 8 ng/egg: 14.6% abnormal embryos, *n* = 41; 16 ng/egg: 30.6% abnormal embryos, *n* = 49). These results were confirmed by a splice-blocking MO, *fgfr2-I4E5* MO (Fig. 1D, 1H, and 1O; 29.6% abnormal embryos, *n* = 27). In order to verify which *fgfr2* variant controlled left-right asymmetry, we designed specific splicing blocking MOs that targeted *fgfr2b* and *fgfr2c*, respectively. According the cDNA sequence data (AB094118 and AB083105), isoform-specific exonic regions could be identified. We further confirmed these regions in *fgfr2b* and *fgfr2c* were exon8 and exon9, respectively, according to annotated zebrafish Zv9 assembly. In order to specifically inhibit the splicing of *fgfr2b* and *fgfr2c*, morpholino target sites were located at E8I8 (*fgfr2b*) and E9I9 (*fgfr2c*). The specificity was verified by RT-PCR analysis and sequencing (supplementary Fig. S1). The predicted translated products of *fgfr2b* and *fgfr2c* had in frame deletions of 13 and 17 amino acids, respectively. The deleted region of Fgfr2c consists of two critical amino acid residues (I350 and Y352) which form hydrophobic groove to interact with Fgf ligands [22]. We found 29.6% (*n* = 375) of embryos to be abnormal in *fgfr2c* morphants (Fig. 1I, 1L, and 1O). However, only 7.4% (*n* = 27) of embryos were observed to be abnormal in *fgfr2b* morphants (Fig. 1J, 1M, and 1O). The laterality of visceral organ was not affected in *fgfr2c* 5-base mismatch (*fgfr2c*-5 mm) morphants (Fig. 1K, 1N and 1O; 3% abnormal embryos, *n* = 133). The phenotype in *fgfr2c* morphants could be rescued with morpholino-resistant *fgfr2c* mRNA (16.9% abnormal embryos, *n* = 118). These results indicate that *fgfr2c* is the major *fgfr2* isoform that regulates the left-right pattern of visceral organs.

### Heart Laterality was Randomized by *fgfr2* Inhibition

**Figure 2:**
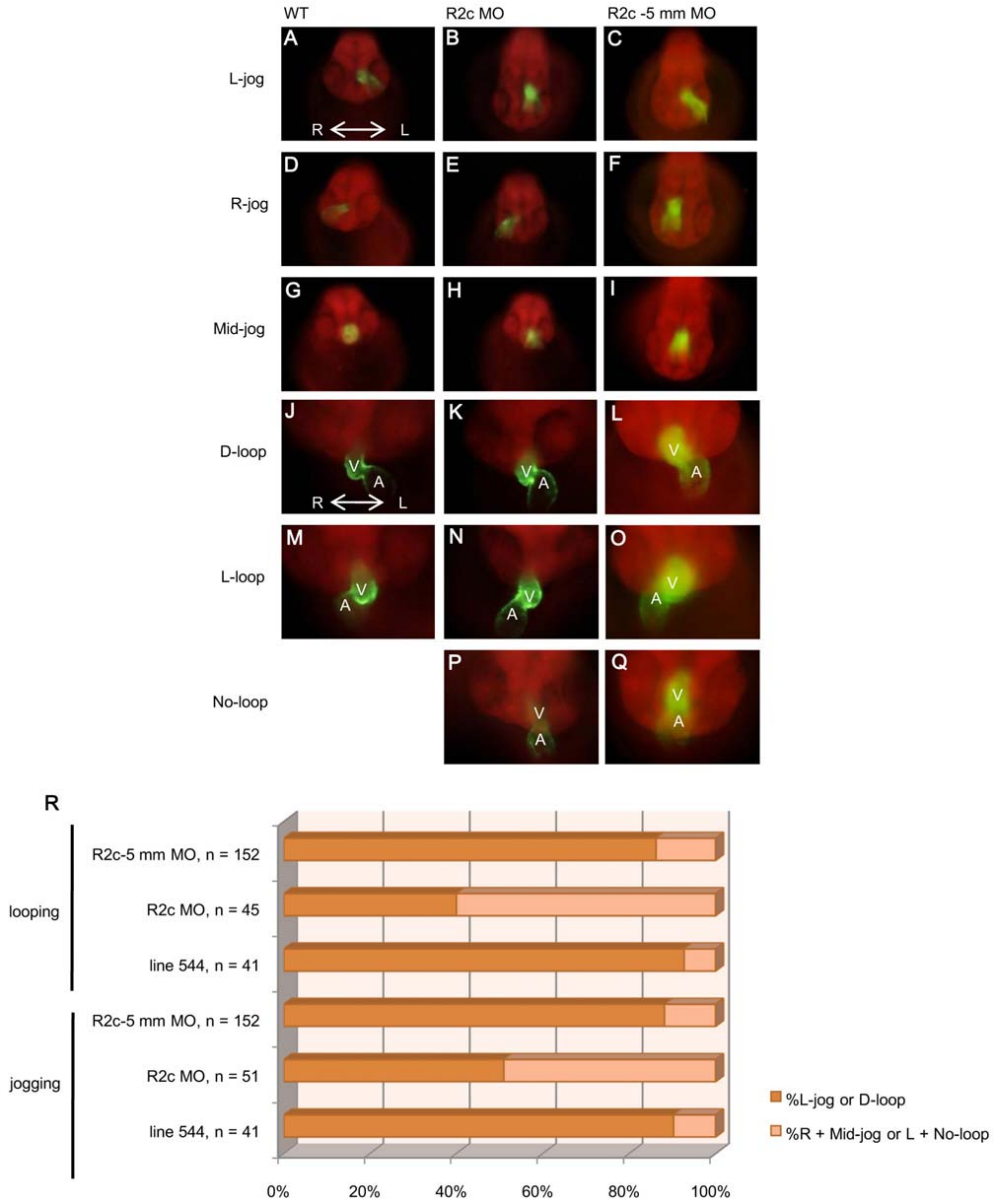
The laterality of heart jogging and looping was randomized in *fgfr2c* morphants. The development of heart was followed using Line 544 (*cmlc2*:EGFP/*β-actin2*-mCherry) transgenic fish. Normal direction of heart jogging was toward left side (A∼C, L-jog). Randomization resulted in abnormal patterns of jogging (D∼I, R-jog and mid-jog). Normal heart looping (J∼L, D-loop) and abnormal heart looping (M∼Q, L-loop and no-loop) were detected in un-injected transgenic line 544 embryos, *fgfr2c* and *fgfr2c*-5 mm morphants. All pictures were ventral-anterior view. The bar charts showed the percentage of different types of heart jogging and looping (R). A: atrium, V: ventricle. Left-right axis was indicated as labeled.

Since the laterality of visceral organs was affected in *fgfr2c* morphants, we wanted to analyze whether the left-right asymmetry of developing heart was also randomized. We examined heart jogging at 30 hpf and looping at 48 hpf using Line 544 (*cmlc2*:EGFP/*β-actin2*-mCherry) fish, in which GFP was specifically expressed in heart. Heart jogging occurred after heart-tube formation, and heart looping took place following heart jogging. In 90.2% (*n* = 41) of the un-injected transgenic line 544 embryos we observed, heart corn migrated toward the left-anterior and formed heart tube (L-jog, Fig. 2A and 2R), and in 92.7% (*n* = 41) of the un-injected line544 embryos we observed, the atrium was located at the left side of ventricle (D-loop, Fig. 2J and 2R). When laterality was disrupted, the direction of heart jogging became random in 9.8% of the un-injected transgenic embryos (heart corn migrated toward the right-anterior, R-jog; heart corn migrated toward mid line, mid-jog, Fig. 2D, 2G, and 2R), and in 7.3% of the un-injected transgenic embryos, the location of the atrium turned toward the right side of ventricle (L-loop, Fig. 2M and 2R). In *fgfr2c* morphants, the numbers of embryo with R-jog or mid-jog increased to 49% (*n* = 51) (Fig. 2E, 2H, and 2R). Furthermore, 60% (*n* = 45) of *fgfr2c* morphants exhibited abnormal heart looping (L-loop or no-loop, Fig. 2N, 2P, and 2R). Embryos injected with *fgfr2c*-5 mm MO were relatively normal in heart jogging (Fig. 2C and 2R; 88.2% L-jog) and heart looping (Fig. 2L and 2R; 86.2% D-loop). According to these results, we conclude that Fgfr2c signaling is required for left-right pattern of heart in zebrafish.

### Expression of *lefty1, spaw* and *pitx2c* were Affected by *fgfr2c* Inhibition

**Figure 3:**
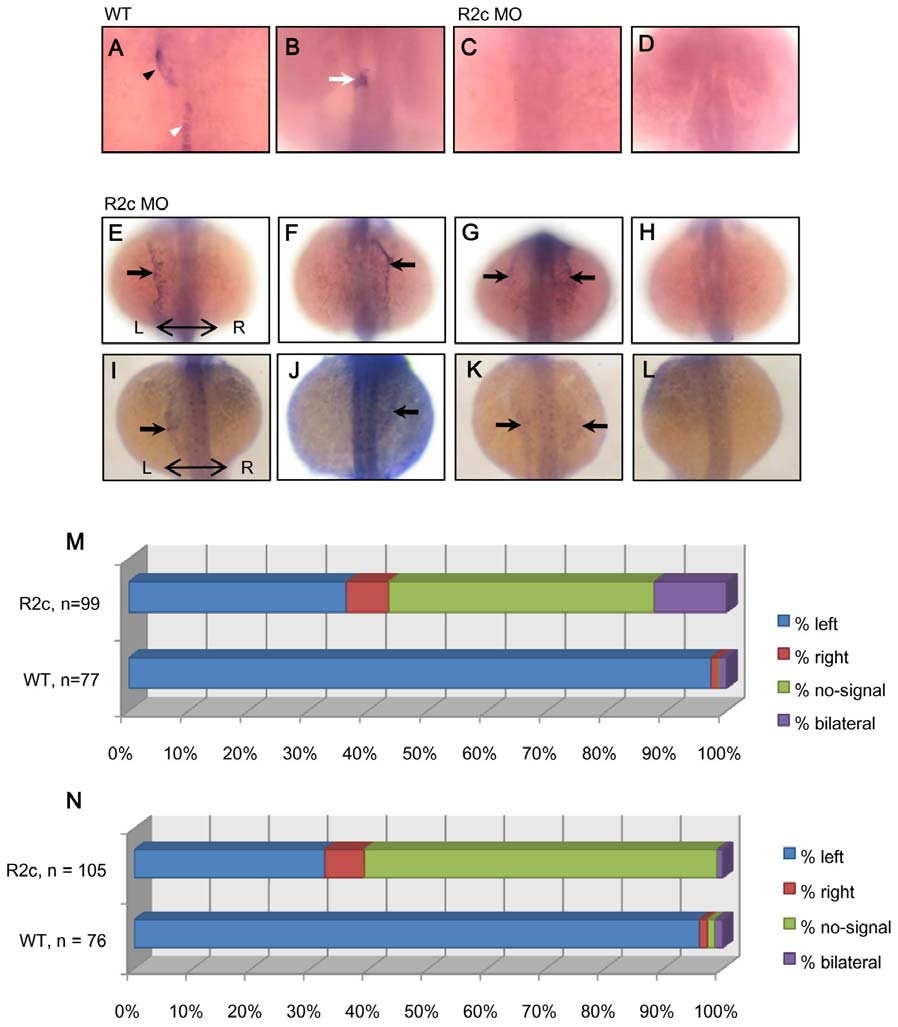
The expression of *lefty1*, *spaw* and *pitx2c* were affected in *fgfr2c* morphants. The *lefty1* was expressed in the mid line (white arrow head), heart primordium (black arrow head) and diencephalon (white arrow) of wild type (A and B) but not expressed in the corresponding regions of *fgfr2c* morphants (C and D). Four different expression patterns of *spaw* and *pitx2c* in LPM (left, right, bilateral and no-signal) were detected in *fgfr2c* morphants (black arrow, E∼L). All pictures were dorsal view. The bar charts showed the percentage of different *spaw* and *pitx2c* expressing pattern in wild type and morphants (M and N). Left-right axis was indicated as labeled.

To verify the molecular mechanism of Fgfr2c signaling in left-right asymmetry, we examined the expression of *lefty1*, *spaw* and *pitx2c*. Normally, *lefty1* was expressed in the notochord as a molecular midline barrier (Fig. 3A) [23]. Eighty percent (*n* = 45) of *fgfr2c* morphants in our experiment showed reduced *lefty1* expression in the midline (Fig. 3C). The *lefty1* gene was also expressed in the left diencephalon of wild type embryos (Fig. 3B) [15]. However, we found that 97.8% (*n* = 46) *lefty1* was not expressed in this region in *fgfr2c* morphants (Fig. 3D). We then examined the expression of the left-side genes, *spaw* and *pitx2c*. For *spaw* expression in wild type embryos, we found that 97.4% (*n* = 77) exhibited left lateral plate mesoderm (LPM) expression; 1.3% exhibited right-side expression; and 1.3% exhibited bilateral expression (Fig. 3M). In the *fgfr2c* morphants, the expression of *spaw* was seen in 36.4% (*n* = 99) in left LPM; 7.1% expressed *spaw* in right LPM, 12.1% exhibited bilateral expression, and 44.4% showed no expression (Fig. 3E–3H, and 3M). The *pitx2c* was expressed in wild type embryos (*n* = 76): left LPM (96.1%); right LPM (1.3%); bilateral LPM (1.3%); and no expression (1.3%) (Fig. 3N). The *pitx2c* expression was affected in *fgfr2c* morphants (left: 32.4%, right: 6.7%, bilateral: 0.9% and no expression: 60%; Fig. 3I–3L, and 3N). These results suggest that the abnormal expression of *spaw* and *pitx2c* may be due to the defective of molecular midline barrier in *fgfr2c* morphants.

### The Ciliogenesis in KV was Affected in *fgfr2c* Morphants

**Figure 4:**
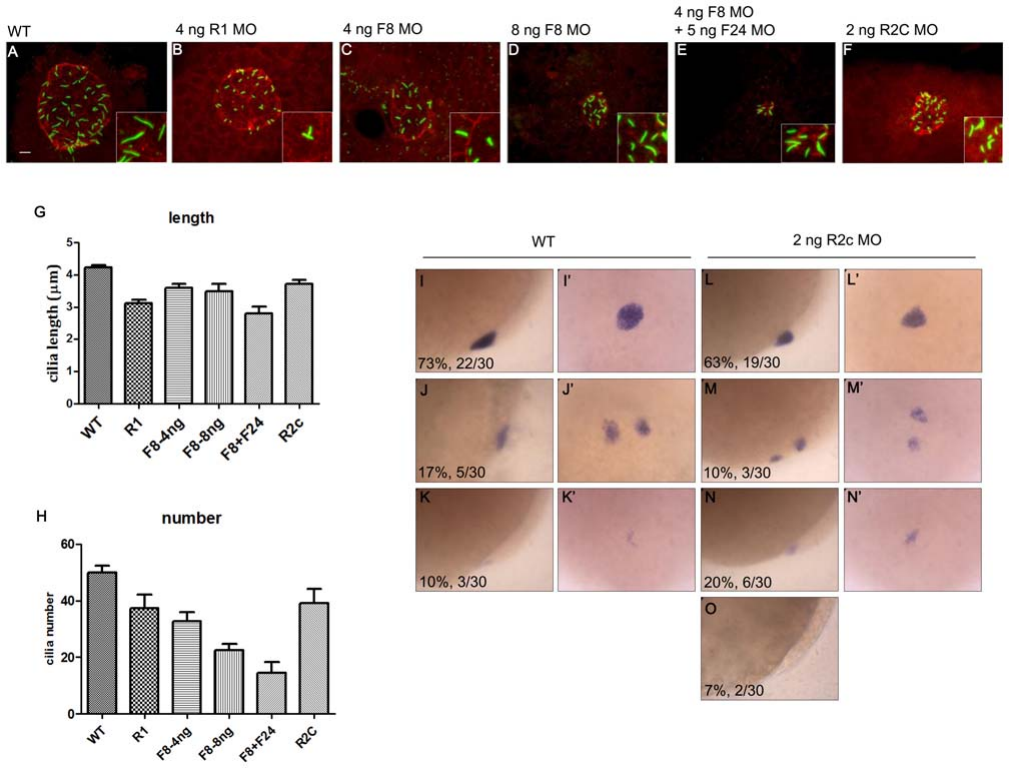
The cilia length was disrupted in *fgfr2c* morphants. The KV and cilia were labeled with antibodies against aPKC (red) and acetylated tubulin (green), respectively, at 10 somite-stage embryos (A∼F). The cilia length was reduced in *fgfr1*, *fgf8* morphants and *fgf8/fgf24* double morphants compared to wild type embryos (A∼E, G). The cilia length was also reduced in *fgfr2c* morphants (F∼G). The cilia number was reduced in *fgfr1*, *fgf8* morphants and *fgf8/fgf24* double morphants compared to wild type embryos (A∼E, H). In *fgfr2c* morphants, the cilia number was not significantly reduced (F, H, 39.2±4.9, *P* = 0.0725). Various expression patterns of *foxj1a* were detected in wild type embryos (lateral view, I∼K; dorsal view, I′∼K′) and *fgfr2c* morphants (lateral view, L∼O; dorsal view, L′∼N′) at 90% epiboly. Error bar, s.e.m. Scale bar: 10 µm.

We next analyzed the cilia of KV. We used acetylated tubulin antibody to detect the cilia morphology of KV in 10 somite-stage embryos (Fig. 4A–4F). The ciliogenesis in KV is important for left–right asymmetry [15], [24], [25]. Fgfr1, Fgf8, and Fgf24 have been reported to regulate the cilia length in KV and other organs [20]. In our results, the cilia lengths were reduced in *fgfr1* morphants (3.1±0.1 µm, 708 cilia, 19 embryos, *P*<0.0001), *fgf8* morphants (3.6±0.1 µm, 752 cilia, 23 embryos for 4 ng/egg, *P*<0.0001; 3.5±0.2 µm, 426 cilia, 19 embryos for 8 ng/egg, *P* = 0.0001), and *fgf8/fgf2*4 double morphants (2.8±0.2 µm, 159 cilia, 13 embryos, *P*<0.0001) compared with wild type embryos (Fig. 4G, 4.2±0.1 µm, 4849 cilia, 97 embryos). The cilia length was also reduced in *fgfr2c* morphants (Fig. 4G, 3.7±0.1 µm, 627 cilia, 16 embryos, *P* = 0.0053) compared with wild type embryos. Furthermore, we counted the number of cilia in the KV of various morphants. The cilia number was reduced in *fgfr1* morphants (37.3±4.9, *P* = 0.0245), *fgf8* morphants (32.7±3.1, for 4 ng/egg, *P* = 0.0006; 22.4±2.4, for 8 ng/egg, *P*<0.0001), and *fgf8/fgf24* double morphants (14.5±3.9, *P*<0.0001) compared with wild type embryos (Fig. 4H, 50.0±2.3). The cilia number was dramatically reduced in *fgf8/fgf24* double morphants, and KV disappeared in 45% (*n* = 20) of *fgf8/fgf24* double morphants. However, the cilia number was not significantly changed in statistics for the *fgfr2c* morphants (Fig. 4H, 39.2±4.9, *P* = 0.0725). Furthermore, KV size was reduced in *fgfr2c* morphants compared with wild type embryos (P<0.001, data not shown). Additionally, we found the expression of *fgfr2* was dynamic. From 95% to 100% epiboly, expression of *fgfr2* could be detected in the marginal YSL (supplementary Fig. S2A∼C). At 5 somite-stage, *fgfr2* was expressed in the area near KV (supplementary Fig. S2D and D′). These results indicate that spatial and temporal expression of Fgfr2 could regulate left–right asymmetry by controlling the length of cilia rather than cilia number. In order to investigate the effects of *fgfr2c* MO specific on dorsal forerunner cells (DFCs), MOs were injected into yolk at 2∼4 hpf [26]. The embryos injected with 4 ng MOs were analyzed by *foxA3* and *cmlc2* whole mount *in situ* hybridization (WISH). The laterality of visceral organs was affected in DFC*^fgfr2c^* ^MO^ morphants (supplementary Fig. S3A, S3B and S3I; 51.8% abnormal embryos, *n* = 180). The heart looping was also affected in DFC*^fgfr2c^* ^MO^ (supplementary Fig. S3E, S3F and S3I; 58.5% abnormal embryos, n = 144). In the control DFC*^fgfr2c^* ^-5 mm MO^ morphants, the laterality was relatively normal (supplementary ; 84.7% for visceral organs, *n* = 163; 84.4% for heart looping, *n* = 162). Taken together, the laterality is disrupted when specifically targeting *fgfr2c* MO in DFCs.

It's known that *foxj1* is important for the motile ciliogenic program [25]. Because the left–right asymmetry is also randomized in *foxj1* morphants [24], we analyzed the expression of *foxj1a* in 90% epiboly embryos. In wild type embryos, *foxj1a* was normally expressed in the KV (Fig. 4I and I′, 73%, *n* = 30). Some embryos (17%) had a scattered expression pattern of *foxj1a* (Fig. 4J and J′), and some (10%) had a reduced signal (Fig. 4K and K′). In *fgfr2c* morphants, the number of embryos with normal expression pattern of *foxj1a* was slightly reduced (Fig. 4L and L′, 63%, *n* = 30). The morphants with abnormal pattern, including scattered expression patterns, reduced signal, and no signal, were increased to 37% (Fig. 4M∼4O). Consistent to our finding, a recent study also indicated that the expression of *foxj1* was downregulated in *fgfr1* morphants. Accordingly, cilia length was also reduced in *fgfr1* morphants [20]. These results indicate that the reason for the reduced cilia length of *fgfr2c* morphants was due to affected expression of *foxj1a*.

## Discussion

In this study, we found that the orientation of asymmetric organs was randomized after knocking down Fgfr2. We further identified *fgfr2c* as the main *fgfr2* variant that regulates the left–right asymmetry. Expression patterns of *spaw*, *pitx2c* and *lefty1* were abnormal in *fgfr2c* morphants. These results indicate that the molecular midline barrier was disrupted and further affects the asymmetric expression of *spaw* and *pitx2c*. Importantly, the cilia length was reduced in the KV of *fgfr2c* morphants.

In mice, chickens, and rabbits, left–right asymmetry requires FGF8 [9], [10], [27]. In zebrafish, Fgf8 signaling can regulate morphogenesis of the KV. KV is lost in about 30% of *ace* mutant embryos, and the laterality of visceral organs, including heart and brain, is also disordered [18]. Recently, some evidence has indicated that Fgf signaling could regulate ciliogenesis in the KV to determine left–right asymmetry [19], [20], [28]. In the KV of *fgfr1* morphants, length of cilia is shorter. This results from the reduction of ciliogenic transcription factors, *foxj1* and *rfx2*, and intraflagellar transport gene *ift88* [20]. In *ace* mutant embryos, the length of cilia is not affected. However, the cilia length is reduced in *ace* mutant embryos injected with *fgf24* MO [20]. These results suggest that *fgf8*, *fgf24*, and *fgfr1* are important for ciliogenesis. In the other hand, two Fgf8 signaling target genes, *ier2* and *fibp1*, have been identified [19]. The cilia number in KV of *ier2* and *fibp1* morphants is also reduced. When *ier2* and *fibp1* mRNA are injected in *fgf8* morphants, the cilia number is restored. Therefore, these two genes can mediate Fgf8 signaling in ciliogenesis and are essential for the establishment of laterality. A recent report indicates that Fgf4 signaling is important for left–right asymmetry [28]. The left–right asymmetry of visceral organs and heart are randomized in *fgf4* morphants. Furthermore, the expression of *lefty1* is absent in the posterior notochord, and the cilia length is reduced despite normal quantities of cilia in *fgf4* morphants. In our studies, reducing cilia length rather than cilia number might result in randomizing the left–right asymmetry in *fgfr2c* morphants. In order to verify which Fgf ligands regulate left–right asymmetry through Fgfr2c, we analyzed the possible synergistic effect of Fgfr2c and the above three mentioned ligands. We examined the heart looping in different low dosage MO combinations, including *fgfr2c*-*fgf4*, *fgfr2c*-*fgf8* and *fgfr2c*-*fgf24* (Supplementary Fig. S4). These preliminary results showed Fgfr2c and Fgf ligands did not have obviously synergistic effect except Fgfr2c-Fgf8. So we suggested that Fgfr2c could functionally interact with Fgf8, whereas Fgf4 and Fgf24 were parallel pathways with Fgfr2c for left–right asymmetry. The disruption of laterality in DFC*^fgf24^* ^MO^ morphants is not known, whereas left–right asymmetry of visceral organs and the heart was affected in DFC*^fgfr2c^* ^MO^ but not in DFC*^fgf4^* ^MO^ morphants [28]. This observation also supports the independence of Fgfr2c and Fgf4 signaling. .

KV formation is very important for left–right asymmetry. The cellular origin of KV is DFCs which migrate at the leading edge of the blastoderm margin [29]. When DFCs is ablated by laser or the KV morphogenesis is disrupted, the expression pattern of left–right asymmetry genes, including *spaw*, *lefty1* and *lefty2,* becomes random [15], [30]. In addition to shorter cilia length, the morphology of KV is changed and its area is reduced significantly in *fgfr2c* morphants (Fig .4F and data not shown). This phenomenon has not been reported in *fgfr1* and *fgf4* morphants [20], [28]. To investigate whether the reduced KV area was due to changes of DFC numbers in *fgfr2c* morphants, we used *casanova* (*cas*) probe to highlight DFCs. Preliminarily we found that the number of *cas* expressed cells was not reduced in the morphants compared to wild type embryos (33.8±0.8 cells in *fgfr2c* moprhants, *n* = 195, and 33.4±0.9 cells in wild type, *n* = 140; *P* = 0.7738). Whether the cell size is affected in the morphants needs to be further examined. Notably, we did find the DFC morphology was obviously different in *fgfr2c* morphants (Supplementary Fig. S5). So, disorganized DFC pattern may cause defects of KV formation. For Fgf related genes in laterality, the *ier2* and *fibp1* have been indicated to affect KV formation starting at the time of DFCs formation [19]. Since these two genes mediate Fgf8 signaling in left–right asymmetry patterning and Fgf8 and Fgfr2c signaling could have functional interaction, we suggest that Fgf8, Fgfr2c and Ier2/Fibp1 may be the same pathway to regulate DFC patterning. Further examination of *fgfr2c* MO specifically targets to DFCs also reveals the disruption of laterality of visceral organs and heart (Supplementary Fig. S3). Therefore, we suggest that Fgfr2 may function cell-autonomously in KV to regulate the organization of DFCs during the laterality establishment. However, the detailed mechanism remains unclear. In addition to KV morphogenesis, the expression of *lefty1* in midline is also important for the left-side expression of *spaw* in LPM. In this study, we found that the expression of *lefty1* in midline was absent in *fgfr2c* morphants. The abnormal expression of *spaw* in the LPM of *fgfr2c* morphants could be due to the loss of *lefty1* in midline. Taken together, we conclude that Fgfr2c signaling controls left–right patterning through regulating the cilia length and controlling the expression of *lefty1* to set up a molecular midline barrier. These suggest that Fgfs have multiple roles in left–right patterning.

## Materials and Methods

### Ethics Statement

All embryos were handled according to protocols approved by the Institutional Animal Care and Use Committee of Tzu Chi University, Hualien, Taiwan (approval ID: 97062).

### Zebrafish

The zebrafish (*Danio renio*) were raised as described in the Zebrafish Book [31]. The AB wild type strain was used for morpholino injection and other experiment. Line 544 (*cmlc2*:EGFP/*β-actin2*-mCherry) was generated by Dr. Chung-Der Hsiao.

### Plasmid Construction

Tol2 kit was used to rapidly assemble expression vectors by two-fragment gateway recombination cloning. The p5E-*β*-actin2 5′ entry clone contains 5.3 kb upstream regulatory sequences of *β-actin2* gene that sufficient to target transgene ubiquitously express. The pME-mCherry middle entry clone contains mCherry fluorescent reporter gene. The p3E-polyA 3′ entry clone contains late polyA sequence from SV40 virus. Finally, p5E-β-actin2, pME-mCherry and p3E-polyA were assembled together with pDestTol2CG2 by LR reaction to create expression vectors of pDestTol2CG2bactin2-mCherry-pA.

### Microinjection

All morpholinos (MOs) were purchased from GeneTools. The sequences of MO used were as follows: *fgfr1* MO, 5′-GCAGCAGCGTGGTCTTCATTATCAT-3′ [32]; *fgfr2*-ATG MO, 5′-CAGAAGCCACCCTCGGGCGAACATC-3′; *fgfr2*-I4E5 MO 5′-GTCGAACCTGGAACGGGAAAGCGTA-3′ [33]; *fgfr2b* MO, 5′-CGCTCCTGCTTTTTTACCTGGTATG-3′; *fgfr2c* MO, 5′-AAGCAGTGGAAGGTGAGTTTATACC-3′; 5-base mismatch for *fgfr2c* MO, 5′-AAcCAcTGcAAGGTcAcTTTATACC-3′; fgf4 MO, 5′- TTTCATACTCACAGATCCGTAAAGC-3′ [28]; *fgf8* MO, 5′-GAGTCTCATGTTTATAGCCTCAGTA-3′ [34]; and *fgf24* MO, 5′-AGGAGACTCCCGTACCGTACTTGCC-3′ [35]. The MOs were injected into the cell at one cell stage. To target MOs to DFCs, MOs were injected into yolk at 2∼4 hpf. To analyze splicing defects after *fgfr2b* and *fgfr2c* MO injection, reverse transcription was carried out using the ImProm-II kit (Promega). The sequences of PCR primers for *fgfr2b* and *fgfr2c* were: 5′-GCTCGGGCATAAACAGCTCGG-3′ (*fgfr2b*-forward), 5′-CGGCAGGTGTGAACACTACGG-3′ (*fgfr2c*-forward), and 5′-CTCCGGCGAGTGGTGATTCTG-3′ (*fgfr2*-reverse). The coding region of *fgfr2c* was amplified from a *fgfr2c* plasmid [36] and subcloned into pCS2+ vector. The primer sequences were: 5′-ACTATCGATATGTTCGCCCGAGGGTGG-3′ (forward), 5′-TGGCTCGAGTCATGTTTTTATGCCGCC-3′ (reverse). Transposase RNA was synthesized *in vitro* by using pCS-transposase plasmid (kindly provided by Dr. Koichi Kawakami) as a template. Capped mRNA was prepared with the mMESSAGE mMACHINE kit (Ambion). To perform rescue experiment, *fgfr2c* mRNA (50 pg/egg) was coinjected with *fgfr2c* MO (2 ng/egg).

### Creation of cmlc2:EGFP/beta-actin2-mCherry transgenic zebrafish

For generation of transgenic zebrafish, we mixed expression constructs of pDestTol2CG2bactin2-mCherry-pA (50 ng/µl) with *in vitro* transcribed transposases mRNA (50 ng/µl) and injected about 1–3 nl DNA solution into the animal pole of one-cell stage embryos. The injected embryos were raised to adulthood and the putative founders were screened according to the green fluorescent signals in the heart of their F1 progenies. We totally identified 10 independent lines out of 89 crosses and used the most robust expression line Tg(cmcl2:EGFP; bactin2:mCherry)^cy1^ for the following experiments.

### Whole mount *in situ* hybridization

The following *in situ* probes were used: *cas* [37], *cmlc2* [38], *fgfr2* [*36*], *foxA3* [39], *foxj1a* [25], *lefty1* and *spaw* (both were provided by Dr. Karuna Sampath, The National University of Singapore), and *pitx2c* [40]. The DIG-labeled probes were generated by *in vitro* transcription using a DIG RNA labeling kit (Roche). For whole mount *in situ* hybridization, DIG-labeled probes were used to hybridize the embryos overnight at 65 or 70°C and then washed with high stringency condition. The embryos were treated with blocking buffer (Roche) and incubated with AP-counjugated anti-DIG antibody overnight at 4°C (1∶8000, Roche). Excess antibody was washed and the embryos were colored with NBT/BCIP.

### Immunofluorescence

Embryos were fixed overnight in 4% paraformaldehyde at 4°C. Fixed embryos were washed with PBST (containing 0.3% TritonX-100) and treated with 10 mM Tris, 1 mM EDTA, 0.05% Tween20 for 5 minutes in 95°C. The embryos were subsequently blocked in PBST containing 4% BSA for one hour. Embryos were incubated in mouse anti-acetylated tubulin (Sigma T-6793, 1∶200) and rabbit anti-aPKC (Santa Cruz sc-216, 1∶100) at 4°C for overnight. After washed with PBST, embryos were incubated in goat anti-mouse Alexa Fluor 488 (Molecular Probes A-11029, 1∶200) and goat anti-rabbit Alexa Fluor 647 (Molecular Probes A-21245, 1∶200) at 4°C for overnight. After washed with PBST, embryos were mounted in SlowFade Gold antifade reagent with DAPI (Molecular Probes S-36938). Embryos were imaged using a LEICA TCS SP2 AOBS confocal microscope. Ciliary length and number were measured using Leica Confocal Software. KV size was analyzed by ImageJ using arbitrary unit. Two-tailed Student's *t*-test was used for analyzing on cilia length and number.

## Supporting Information

Figure S1 **The effects of** ***fgfr2b*** **and** ***fgfr2c*** **specific MOs.** (A and B): Blue arrows were primer sites for RT-PCR to detect the splicing products. Red thick lines were MO target sites. Injection of *fgfr2b* (4 ng per embryo) or *fgfr2c* (1, 2 or 4 ng per embryo) MOs caused partial deletion of exon 8 (b) and exon 9 (c), respectively, that had been confirmed by sequencing. The original splice donor sites were blocked and cryptic splice donor sites in exon8 and exon9 were activated (indicated by bottom red lines) by the corresponding MOs. The partial cDNA sequences were shown (exon7, 8, 10 and 11 for *fgfr2b* and exon 7, 9 10 and 11 for *fgfr2c*). Underline indicated the primer sequence. The deleted regions were highlighted.
(EPS)

Figure S2 **Expression pattern of** ***fgfr2*** **.** The expression of *fgfr2* was detected in marginal YSL (A∼C, arrow, 95%∼100% epiboly) and in the area near KV (arrowhead in D′, 5 somite-stage). Boxed area shown in panel D is enlarged in panel D′.
(EPS)

Figure S3 **The effects of** ***fgfr2c*** **MO specific on DFCs.** The normal expression pattern of *foxA3* in liver (l) and pancreas (p) was shown in DFC*^fgfr2c^* ^MO^ and DFC*^fgfr2c^* ^-5 mm MO^ morphants (A and C). Abnormal pattern of visceral organs was also observed in these embryos (B and D). The development of heart was examined using *cmlc2* probe (E∼H). Normal (E and G) and abnormal heart looping (F and H) can be observed in both morphants. The bar charts showed the percentage of embryos with different expression distribution of *foxA3* or *cmlc2* in both morphants (I). Panel A to D were dorsal view and panel E to H were ventral-anterior view. A: atrium, V: ventricle.
(EPS)

Figure S4 **The percentage of abnormal heart looping in** ***fgfr2c*** **and** ***fgf*** **ligand morphants.** In order to test the synergistic effect of Fgfr2c and Fgf ligands (Fgf4, Fgf8 and Fgf24), different combinations of low dosage *fgfr2c* MO and *fgf* MOs were injected into Line 544. Double morphants of *fgfr2c* and *fgf4* (2, 1 or 0.5 ng/embryo for *fgfr2c* MO; 34, 22.5 or 12 ng/embryo for *fgf4* MO) did not have synergistic effect on the abnormal heart looping, including L-loop and no loop pattern (A). Co-injection with *fgfr2c* MO (0.5 ng/embryo) and *fgf24* MO (1.25 ng/embryo) also did not greatly increase the abnormal percentage (B). In contrast to above results, a synergistic effect was detected in *fgfr2c*-*fgf8* double morphants (0.5 ng/embryo for *fgfr2c* MO and 1 ng/embryo for *fgf8* MO; C).
(EPS)

Figure S5 **The** ***cas*** **expression pattern in** ***fgfr2c*** **morphants.** Embryos at 90% epiboly were stained with *cas* probe for labeling DFCs. The morphology of normal DFC cluster in wild type was shown in panel A (79%, *n* = 140). The mild and severe disorganization of DFC pattern could also be detected. However, the percentages of abnormal pattern were increased in *fgfr2c* morphants (B and C, 56.9%, *n* = 195).
(EPS)
